# Human–Animal Therapeutic Relationships: Communication, Welfare and Interpretative Asymmetry

**DOI:** 10.3390/ani16162509

**Published:** 2026-08-12

**Authors:** Waldemar J. Grzegorzewski, Hanna Mamzer, Maria Kuchtar

**Affiliations:** 1 Faculty of Biology, Nature Protection and Sustainable Development, University of Rzeszów, 35-601 Rzeszów, Poland; mkuchtar@ur.edu.pl; 2 Faculty of Sociology, Adam Mickiewicz University in Poznań, 60-568 Poznań, Poland

**Keywords:** human–animal relationships, non-human animals, animal welfare, human–animal studies, interspecies communication, interpretative asymmetry, animal-assisted interventions, equine-assisted interventions, affective labour, anthropomorphism

## Abstract

Human–animal therapeutic relationships are commonly evaluated through their effects on human psychological, emotional and social well-being, whereas the experiences and welfare of non-human animals remain less consistently examined. This narrative interdisciplinary review analyses such relationships through interpretative asymmetry, integrating human–animal studies, ethology, animal welfare science and biological approaches to interspecies communication. Selected cultural examples are used only as illustrative boundary narratives. Interpretative asymmetry is defined not as a statistically measured variable, but as a conceptual and structural imbalance in interpretative authority, communicative access and agency. Humans define therapeutic goals, organize interaction and attribute meanings such as trust, reciprocity or support to non-human animal behaviour. Non-human animals respond through species-specific sensory, behavioural and physiological mechanisms that may indicate affiliation, habituation, stress regulation, inhibition or conflict avoidance. Animal-assisted interventions and animal-assisted therapy (AAI/AAT) should therefore be assessed through human benefit and animal welfare indicators, including workload, rest, agency, withdrawal opportunities and handler competence. Dogs and horses are discussed as applied contexts in which these asymmetries become visible, whereas wild-animal narratives are treated as boundary examples of projection and romanticization. Recognizing non-human animals as active, welfare-sensitive participants is essential for ethical therapeutic practice.

## Simple Summary

Therapeutic relationships between humans and non-human animals are usually discussed in terms of the benefits they provide for people. Contact with dogs, horses or other non-human animals may reduce stress, support emotional regulation, increase motivation and facilitate social contact. However, the same interactions also affect the non-human animals involved, whose experiences are often less visible and must be inferred from behaviour, physiology and context. This review examines therapeutic human–animal relationships as asymmetrical interspecies interactions. We use the term “interpretative asymmetry” to describe a situation in which humans define the goals of the interaction, organize its space and timing, and assign emotional meanings to non-human animal behaviour, whereas the non-human animal participates through species-specific sensory, behavioural and physiological responses that are only accessible indirectly to humans. We argue that animal-assisted interventions should not treat non-human animals merely as therapeutic instruments. They should be understood as active, welfare-sensitive participants whose agency, workload, need for rest and possibility of withdrawal must be included in ethical and practical decision-making. Selected cultural examples are used only as boundary narratives illustrating risks of projection and romanticization, not as evidence of animal-assisted therapy.

## 1. Introduction

For clarity, throughout this article, the term “animal” refers to non-human animals unless otherwise specified, and “human–animal relationship” refers to a relationship between humans and non-human animals. The use of non-human animals in activities aimed at supporting human mental health and well-being can be traced back at least to the eighteenth century and includes some of the earliest attempts to introduce animals into care institutions and psychiatric hospitals [1,2]. Non-human animals were expected to stimulate patients emotionally while simultaneously engaging them in caregiving and therapeutic activities [2]. These relationships were often built around everyday practices such as feeding, grooming and caring for animals. Both farm animals and companion animals were involved in such interventions.

Despite their presence in therapeutic settings, non-human animals were primarily regarded as instruments supporting human well-being, and their participation was interpreted mainly through the benefits they provided to people. This does not imply that they were necessarily treated poorly or that their needs were entirely neglected. However, it is important to recognize that the dominant positivist model of nineteenth-century science did not encourage viewing non-human animals as subjects possessing their own emotional, social and species-specific needs [3,4].

Assessments of animal welfare at that time focused largely on physical health and maintaining animals in a condition that enabled them to perform designated functions. Much less attention was paid to issues such as agency, environmental control, emotional security or sensory overload, all of which are now regarded as important components of animal welfare [4,5].

Consequently, even the earliest attempts to involve non-human animals in therapeutic activities reveal a marked asymmetry in human–animal relationships, in which human needs and well-being occupied a central position, while the perspective of the non-human animal remained largely outside the scope of scientific and ethical reflection. This asymmetry concerned not only the organization of therapeutic interactions but also the very understanding of communication between humans and other species.

**Figure 1:**
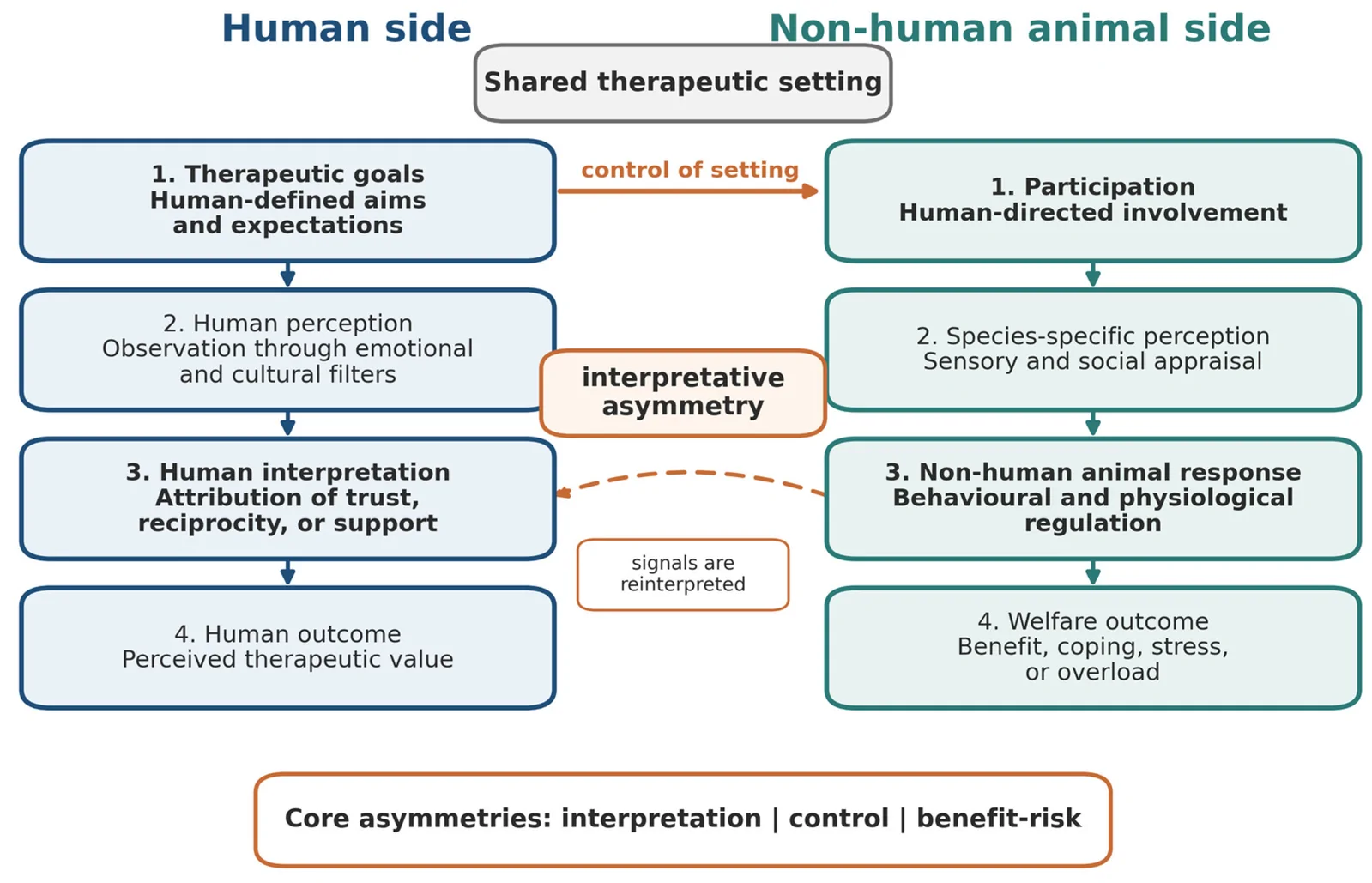
Interpretative asymmetry in therapeutic human–animal relationships.

In this review, interpretative asymmetry is understood not as a statistically measured variable, but as a conceptual and structural feature of therapeutic human–animal relationships. It refers to the unequal distribution of interpretative authority, communicative access and agency between humans and non-human animals. Humans define the therapeutic goals of the interaction, organize its spatial and temporal conditions and assign meaning to the animal’s behaviour. Non-human animals, in contrast, participate through species-specific behavioural, sensory and physiological responses that remain only accessible indirectly to human interpretation. Figure 1 visualizes this asymmetry as a parallel but unequal system in which a shared therapeutic setting is filtered through different positions of control, interpretation and welfare visibility.

Presented above is a conceptual diagram developed by the authors to synthesize the argument presented in the text. The model shows that the therapeutic setting is shared, but the goals, timing and evaluative frame are primarily human-defined. Non-human animal responses return to the relationship mainly as human interpretation, while welfare consequences must be inferred from behavioural, physiological and contextual indicators.

Humans often interpret non-human animal behaviour through the lens of their own emotional, social and cultural needs, sometimes overlooking biological and species-specific mechanisms of communication [6,7]. Ethological and neurobiological studies indicate that non-human animals participating in therapeutic relationships are not passive components of a therapeutic environment but active participants in social interactions, responding to human signals through both behavioural and physiological pathways [8,9]. Research has shown that interactions with humans may influence stress levels, activity of the hypothalamic–pituitary–adrenal axis and physiological processes associated with social attachment [10,11]. At the same time, non-human animals may experience sensory overload, chronic tension or motivational conflicts that often remain unnoticed by untrained observers [12,13].

These observations call for a reconsideration of therapeutic human–animal relationships not only in terms of their effectiveness for humans but also with regard to animal welfare, communication and subjectivity. Of particular importance is the question of whether such relationships are genuinely reciprocal or whether they remain structures shaped predominantly by human interpretations of the needs and behaviours of other species [3,14,15].

The aim of this review is therefore to analyze therapeutic human–animal relationships as asymmetrical interspecies interactions in which human therapeutic benefit, animal welfare, communication and interpretation intersect. Rather than evaluating the clinical efficacy of animal-assisted interventions and animal-assisted therapy (AAI/AAT), the article examines how therapeutic meanings are constructed by humans and how these meanings may obscure the biological and welfare-related needs of the non-human animals involved. This interdisciplinary approach may contribute to a more comprehensive understanding of both the therapeutic potential of these relationships and their consequences for the animals participating in them.

## 2. Methods

This article adopts a narrative and interdisciplinary literature review approach. The analysis encompasses publications addressing animal-assisted interventions and animal-assisted therapy (AAI/AAT), animal welfare, interspecies communication, cognitive ethology and human–animal studies. In addition, selected documentary and cultural sources depicting human–animal relationships were included as illustrative material for conceptual analysis.

The literature search was conducted using Web of Science, Scopus and Google Scholar. Searches were conducted between January and April 2026, without restrictions regarding publication year because of the conceptual and interdisciplinary nature of the topic. Search terms included: animal-assisted therapy, animal-assisted intervention, therapy dog welfare, equine-assisted interventions, animal welfare, interspecies communication, human–animal bond, anthropomorphism, cognitive ethology, therapeutic relationships and cross-species interaction. Because the manuscript integrates biological and humanistic perspectives, selected sources from sociology, philosophy of interspecies relations, animal studies and human–animal studies were also considered. The search was targeted and iterative rather than exhaustive. Search outputs were therefore not treated as a numerical sampling frame; instead, records were screened by title, abstract and full text for relevance to the conceptual aim of the review, and additional sources were identified through backward and forward citation tracking from key works.

Included sources were peer-reviewed articles, scholarly books and book chapters addressing at least one of the following dimensions: (i) therapeutic or supportive human–animal relationships; (ii) animal welfare, stress, agency or workload in AAI/AAT; (iii) interspecies communication and ethological interpretation; or (iv) theoretical analyses of human dominance, affective labour and anthropocentric interpretation. Sources focused exclusively on human clinical outcomes were used only when they were necessary for contextualizing AAI/AAT and were not treated as sufficient evidence for animal welfare. Studies and conceptual works that allowed integration of human benefit with animal welfare and communication were prioritized.

Due to the conceptual character of this work, the objective was not to perform a systematic review or meta-analysis, but to identify key interpretative challenges associated with therapeutic human–animal relationships and to integrate humanistic, ethological and biological perspectives. The review was therefore not designed according to PRISMA guidelines and does not provide quantitative synthesis, effect-size estimates or statistical verification of asymmetry.

The selected documentary and cultural examples were not treated as empirical evidence of animal-assisted therapy or animal welfare outcomes. They were included only as boundary narratives illustrating how human emotional needs, cultural expectations and symbolic interpretations may shape understandings of interspecies relationships. This distinction is important because the central object of analysis is not the clinical efficacy of the examples, but the interpretative mechanisms through which humans assign meaning to non-human animal behaviour. All figures are original conceptual diagrams prepared by the authors to synthesize the arguments emerging from the literature reviewed in this article; they are not adaptations of any single published figure.

## 3. Human–Animal Therapeutic Relationships: Historical and Conceptual Background

The origins of using non-human animals to support human health and well-being were largely rooted in the intuitive belief that contact with other living beings could generate positive emotional and social outcomes. As early as the eighteenth and nineteenth centuries, observers noted that the presence of animals appeared to promote patient engagement, reduce social isolation and improve daily functioning among individuals residing in care institutions and psychiatric facilities [1,2]. Human–animal relationships gradually came to be viewed not only in terms of work or utility but also as a potential source of psychological support.

The development of contemporary animal-assisted interventions, however, was primarily driven by a focus on outcomes achieved by humans. Non-human animals were generally regarded as therapeutic facilitators, enhancing well-being or motivating patients to engage in specific activities. Consequently, an anthropocentric perspective dominated the field, with improvements in human welfare serving as the principal criterion for evaluating therapeutic effectiveness [16].

Only with the emergence of modern ethology, cognitive ethology and animal welfare science did growing attention begin to be directed toward the welfare of non-human animals involved in therapeutic settings and toward their social and emotional subjectivity [4,5]. Increasingly, researchers have emphasized that non-human animals participating in therapeutic interactions are not merely passive “tools” of therapy but living organisms with their own needs, communication strategies and individual emotional responses.

Human–animal relationships have also historically been shaped by the coexistence of affection, care, control and domination. The asymmetry analyzed in this review is therefore not only a matter of possible misreading of behaviour, but also a structural feature of relationships in which humans define spaces, roles, acceptable behaviours and the functions assigned to other species. Tuan’s analysis of dominance and affection in the making of pets is particularly relevant here, because it shows that emotional closeness to non-human animals may coexist with strong human control over their bodies, movements and social roles [17]. This concern also resonates with political approaches that question whether domesticated animals should be regarded only as resources or as members of shared human–animal communities [18]. Therapeutic relationships therefore do not emerge in a neutral space. They are situated within a broader history of human-defined animal functions, where the animal may be loved, protected and valued, while still remaining subject to human decisions about participation, meaning and use.

Contemporary approaches to human–animal relationships increasingly move away from the language of “using animals” and instead emphasize the significance of interspecies relationships themselves. This shift influences not only the language employed to describe human–animal interactions but also the ways in which non-human animal needs and behaviours are understood. As Wittgenstein [19] argued, the manner in which reality is described influences how it is understood. Consequently, the way humans describe non-human animals and their relationships with them shapes interpretations of animal needs, emotions and behaviours.

At this point, the very notion of a human–animal relationship becomes particularly important. The term “relatio” refers both to a connection between entities and to the interpretation of shared experiences. In interspecies relationships, these dimensions are closely intertwined because humans inevitably interpret non-human animal behaviour through the lens of their own experiences and relational frameworks [3,15,20].

Research on interspecies communication suggests that many human interpretations of non-human animal behaviour are vulnerable to anthropomorphic bias and misinterpretation of biological signals. Humans often perceive tolerance of contact, the absence of avoidance behaviour or affiliative actions as evidence of reciprocity, whereas from an ethological perspective such behaviours may result from habituation, conflict-avoidance strategies, learned tolerance or environmental dependence [7,21].

In animal-assisted interventions, this structural asymmetry may also take the form of affective labour performed by non-human animals for human benefit. Such labour includes more than physical presence or trained behaviour: it may involve providing comfort, facilitating emotional regulation, creating a sense of safety and supporting social interaction. These contributions are often valued through the improvement observed in humans, whereas the effort, tolerance, fatigue, agency or welfare cost on the animal’s side may remain less visible. This does not mean that AAI/AAT is necessarily exploitative or that non-human animals cannot experience positive interactions with humans. Rather, it means that therapeutic benefit is produced within an asymmetrical relationship in which one party defines the aims and evaluates success, while the other participates through behavioural and physiological responses that require careful welfare-sensitive interpretation [22,23,24].

As a result, contemporary understandings of therapeutic human–animal relationships require the integration of humanistic reflection with biological and ethological knowledge. Such an approach not only facilitates a more comprehensive assessment of potential therapeutic benefits for humans but also highlights the implications of these relationships for animal welfare, communication and functioning.

## 4. Communication, Interpretation, and Asymmetry in Interspecies Relationships

One of the central challenges in the analysis of therapeutic human–animal relationships concerns interspecies communication and the limitations associated with interpreting signals produced by organisms belonging to different biological species. Humans frequently interpret non-human animal behaviour through reference to their own emotional, social and cultural experiences, a process that may lead both to anthropomorphism and to misinterpretation of the actual meaning of animal behaviour [6,7]. Table 1 provides a deliberately concise set of examples in which common human readings of behaviour should be checked against alternative or overlooked ethological interpretations.

Table 1 should be read as an interpretative caution rather than as a diagnostic list. It does not imply that human interpretations are necessarily false, nor that ethological interpretations provide complete access to the non-human animal’s subjective experience. Rather, it shows that the same behaviour may carry different meanings depending on context, prior learning, motivational state and the degree of control retained by the animal. The central problem is therefore not the impossibility of interspecies understanding, but the risk of treating human emotional interpretation as sufficient evidence of animal comfort, reciprocity or consent.

This issue becomes particularly important in relationships described as therapeutic because their effectiveness is typically evaluated primarily from the human perspective. Improvements in patient well-being, reductions in stress, increased feelings of safety or greater motivation are often interpreted as evidence of a positive and reciprocal relationship between humans and non-human animals. Far less attention is devoted to understanding what these interactions mean for the non-human animals themselves and how they may perceive the presence and behaviour of humans.

**Figure 2:**
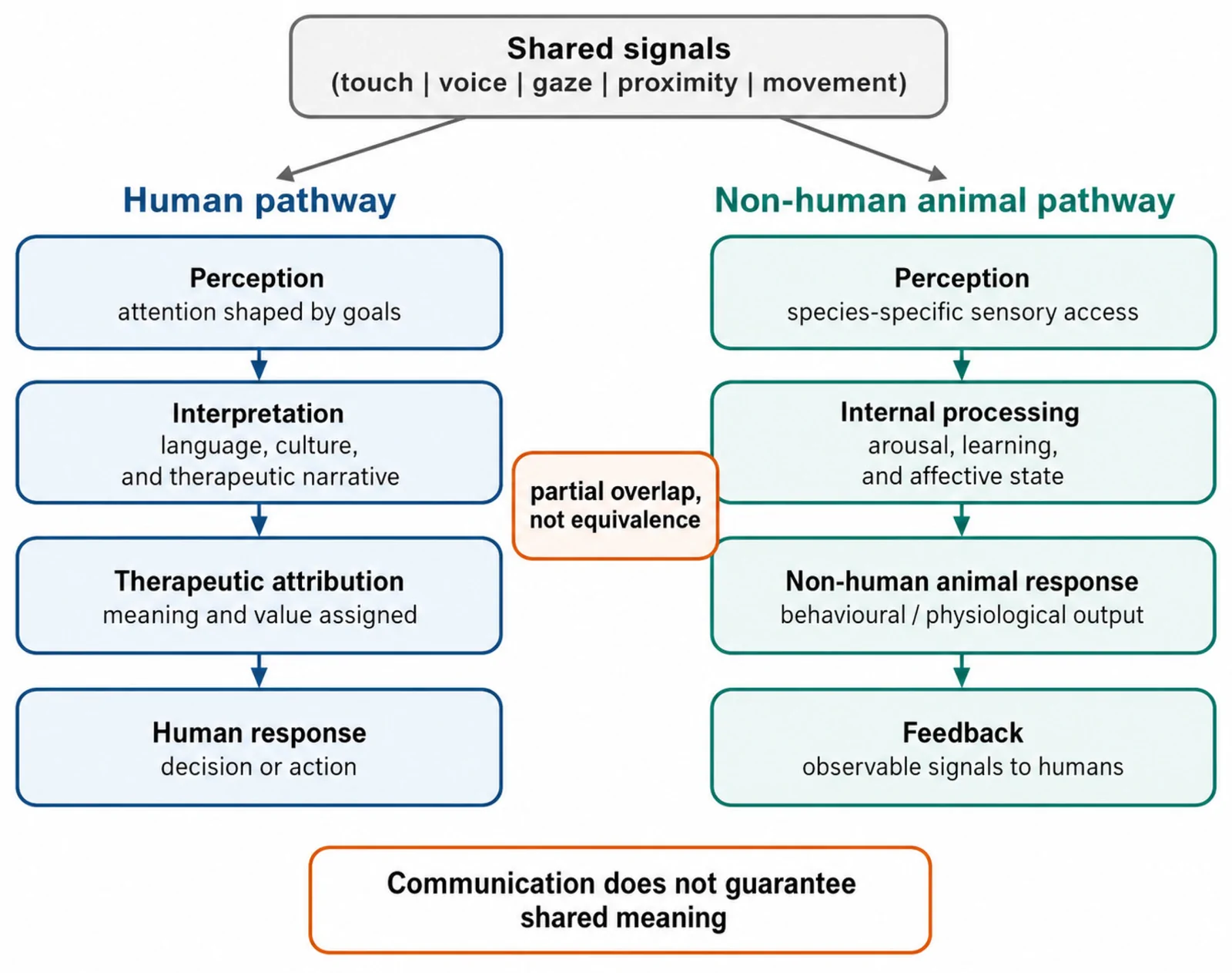
Biological and interpretative levels of cross-species communication in therapeutic interactions.

Contemporary ethology demonstrates that communication between organisms relies on complex, species-specific signalling systems involving visual, acoustic, chemical and tactile stimuli [25]. The interpretation of such signals depends on perceptual capacities as well as environmental and social context. Consequently, humans and non-human animals may participate in the same social situation while processing it through different sensory, cognitive, emotional and relational filters. Figure 2 summarizes this logic by separating the shared signal from the non-equivalent interpretative pathways through which it is processed on both sides of the interaction.

Conceptual diagram developed by the authors. The model separates shared signals from the pathways through which humans and non-human animals process them. Human meaning-making is shaped by language, culture and therapeutic goals, whereas the non-human animal’s experience is accessible only indirectly through behavioural and physiological outputs. The same interaction may therefore be behaviourally shared without being semantically equivalent.

In companion animals, an additional challenge arises from their long-term adaptation to human-created environments. Dogs, cats and horses learn to function within human social worlds; however, this does not imply complete compatibility between human and non-human animal communication systems. In many situations, behaviours interpreted by humans as signs of attachment or acceptance may instead represent adaptive strategies that enable non-human animals to reduce conflict or avoid stressful situations [12,13].

Communicative asymmetry also stems from the fact that humans occupy a dominant interpretative position. Humans define the meaning of the relationship, determine its therapeutic goals and interpret non-human animal behaviour. Non-human animals lack the ability to communicate their experiences linguistically or to challenge human interpretations of their behaviour. Consequently, therapeutic relationships often operate within a one-sided interpretative framework in which human needs and understandings remain the primary reference point.

Neurobiological and ethological research further demonstrates that non-human animals actively respond to human signals and may experience physiological changes associated with social interactions. Human contact can influence cortisol concentrations, autonomic nervous system activity and oxytocin secretion [9,11]. Such responses should not automatically be interpreted as evidence of emotional reciprocity in the human sense of the term. Rather, they may reflect adaptive biological mechanisms associated with stress regulation, environmental predictability or affiliative processes.

For this reason, analyses of therapeutic human–animal relationships require interpretative caution and recognition of the biological limitations of human understanding of interspecies communication. A particularly important question is whether humans genuinely recognize the needs and experiences of non-human animals or whether they primarily interpret them through their own assumptions regarding attachment, reciprocity and emotional connection.

## 5. Animal Welfare and the Problem of the “Silent Participant” in Therapeutic Relationships

Discussions concerning animal-assisted interventions increasingly emphasize the need to consider the welfare of non-human animals involved in therapeutic relationships. For many years, the dominant perspective focused almost exclusively on benefits experienced by humans. Non-human animals were analyzed primarily as components of the therapeutic environment, factors supporting treatment processes or improving human psychological well-being. Far less attention was paid to the consequences of these relationships for the non-human animals themselves.

This issue becomes particularly apparent when non-human animals are regarded as active participants in social interactions while simultaneously lacking the ability to express their own perspectives in ways that humans can unequivocally understand. Non-human animals involved in therapeutic settings become, in a sense, “silent participants”: present, responsive and capable of influencing human emotions and behaviours, yet interpreted largely through human assumptions concerning attachment, trust and reciprocity.

Contemporary animal welfare science emphasizes that animal welfare cannot be reduced solely to appropriate physical conditions or the absence of disease. Equally important are opportunities for environmental control, predictability of social situations, a sense of security and the ability to express species-specific behavioural needs [5,26]. Within this framework, therapeutic relationships may serve both as sources of positive experiences and as potential sources of emotional or sensory overload. In non-human animals exposed to intensive social interactions, such overload may manifest through increased self-soothing behaviours, excessive panting, yawning, avoidance, reductions in exploratory activity or behavioural inhibition. Selected potential benefits and risks associated with animal participation in therapeutic relationships are summarized in Table 2.

Table 2 is intended as a compact welfare framework rather than a universal protocol. The same therapeutic context may be beneficial, neutral or harmful depending on the species, individual temperament, previous experience, session length, environmental predictability, handler competence and the degree of control retained by the non-human animal. In practice, the domains listed in the table should be assessed together rather than in isolation, because workload, autonomy, social contact and recovery jointly shape welfare outcome.

An important challenge concerns the interpretation of non-human animal behaviour. Humans often perceive calm behaviour as an indication of comfort or acceptance of a given situation. From an ethological perspective, however, similar behavioural patterns may have different biological meanings. Avoidance of contact, behavioural inhibition, reduced behavioural expression or excessive submissiveness may represent conflict-reduction strategies, stress responses or forms of adaptation to a human-controlled environment [12,13].

In therapy dogs, welfare assessment should therefore combine behavioural observation with physiological and contextual measures whenever feasible. Reviews of therapy dog welfare emphasize that cortisol alone is insufficient, and that welfare should be assessed through combined indicators, including body posture, avoidance, affiliative motivation, handler–dog interaction, heart rate or heart rate variability, rest opportunities and the broader environmental context [27]. This is particularly important in sessions involving children, hospital environments or unfamiliar participants, where human benefit may be highly visible while canine fatigue, inhibition or subtle avoidance remains overlooked.

Equine-assisted interventions raise related but species-specific welfare concerns. Horses may participate in groundwork, therapeutic riding, hippotherapy or equine-assisted psychotherapy, each involving different levels of human contact, physical load and environmental demand. Stress assessment in horses requires attention to behaviour, autonomic and endocrine indicators, posture, locomotor comfort, recovery time and the cumulative effects of repeated sessions [28]. The therapeutic benefit perceived by the human participant should therefore not be treated as sufficient evidence that the horse experiences the interaction positively.

Neurobiological studies further demonstrate that social interactions between humans and non-human animals may influence systems involved in stress regulation and affiliative processes. Social contact may modulate cortisol secretion, oxytocin release and autonomic nervous system activity in both humans and non-human animals [10,11]. However, this does not automatically imply that the relationship is reciprocal in the human sense of emotional bonding. Physiological responses may also reflect adaptive biological mechanisms associated with environmental predictability, tension reduction or social learning.

A further important issue is the asymmetry of agency. Humans organize therapeutic relationships, define their objectives and evaluate their effectiveness. Non-human animals generally have limited opportunities to influence the conditions of participation or to withdraw from interactions in a manner comparable to humans. For this reason, increasing attention is being paid to the need to consider individual characteristics, including temperament, previous experiences and adaptive capacities, when planning therapeutic interventions [29,30].

**Figure 3:**
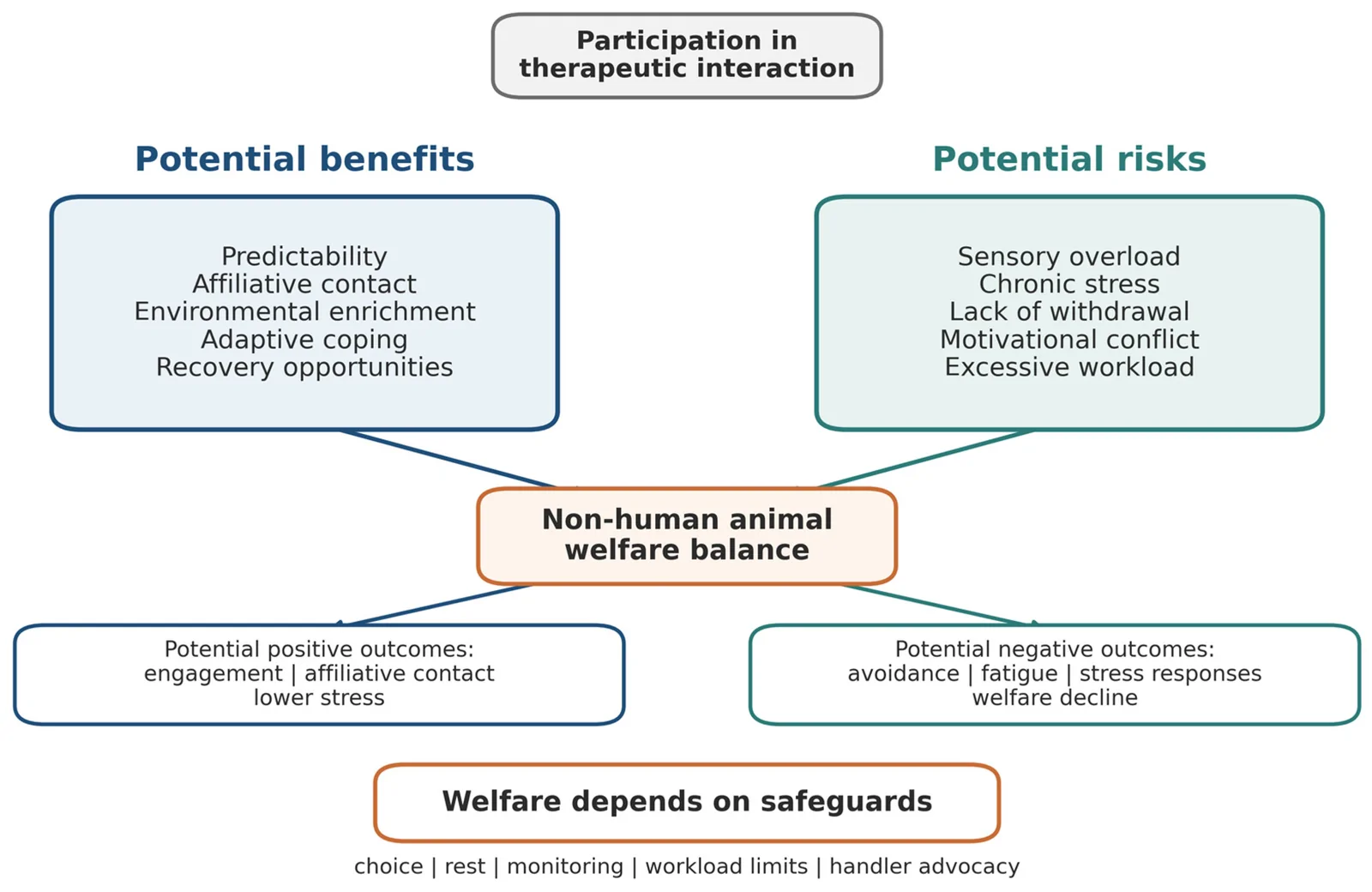
Animal welfare balance in therapeutic relationships.

As a consequence, the analysis of therapeutic human–animal relationships requires moving beyond the simplified view of animals as passive “tools of therapy”. Non-human animals remain living organisms with their own biological, emotional and social needs, and their welfare should be regarded as an integral component of the therapeutic relationship rather than merely a prerequisite for successful therapeutic outcomes in humans. Figure 3 therefore presents welfare as a dynamic balance: the same therapeutic interaction may be supportive, neutral or burdensome depending on predictability, control, rest, workload, handler competence and the possibility of withdrawal.

Conceptual diagram developed by the authors. The model summarizes supportive conditions and risk conditions that may influence the welfare outcome of a non-human animal involved in animal-assisted interventions or therapy. Predictability, choice, rest, species-appropriate interaction and attentive handling may support welfare, whereas overload, forced proximity, inadequate recovery, misread signals and limited control may shift the same interaction toward stress or welfare decline.

## 6. Trust, Control, and Agency in Interspecies Therapeutic Relationships

Trust constitutes one of the fundamental elements in the formation of social relationships, both among humans and among representatives of different species. In the context of therapeutic human–animal relationships, the concept of trust acquires particular significance, as it frequently serves as the basis for interpreting a relationship as safe, supportive and emotionally meaningful.

Classical psychological and sociological concepts describing the formation of social bonds are increasingly being applied to interspecies relationships. This includes, among others, Bowlby’s attachment theory [31] as well as the concept of basic trust developed by Winnicott [32] and Fromm [33]. In human–animal relationships, trust is often understood as the ability to predict the behaviour of the other party, a sense of security and the possibility of developing a stable emotional bond.

In companion animals, trust-based relationships may contribute to reduced stress and increased predictability of the social environment. Studies indicate that stable and calm interactions between humans and animals may promote affiliative processes and emotional regulation on both sides of the relationship [9,10]. Studies of social referencing show that dogs use humans as a source of social information in situations involving uncertainty or environmental novelty [34,35]. However, this does not automatically imply an identical interpretation of a situation by both parties.

This issue becomes particularly important when humans interpret animal behaviour through their own emotional categories. Behaviours indicating predictability or tension reduction may be perceived as evidence of a deep emotional bond, whereas from a biological perspective they may primarily reflect social learning processes, monitoring of the caregiver or adaptation to a human-controlled environment. In dogs, such behaviours may include persistent following of a caregiver, maintaining close social proximity, head turning or nose licking, which, depending on the context, may indicate either affiliation or attempts to reduce social tension.

Studies employing the Strange Situation Test have demonstrated that many dogs exhibit behavioural patterns resembling attachment observed in human children, treating their caregiver as a so-called secure base during environmental exploration [36,37]. These findings indicate the existence of strong social bonds between humans and dogs; however, they do not determine whether such relationships should be interpreted as equivalent to human emotional experiences.

Human–animal relationships also reveal a tension between trust-building and the need for control. On the one hand, therapeutic practice emphasizes cooperation, positive communication and emotional security. On the other hand, the human participant, therapist or handler usually controls the time, space, intensity and termination of the interaction. Such control may be necessary for safety, yet it also reveals an asymmetry of agency: the human defines the conditions of trust, while the non-human animal may experience the situation primarily as predictable but constrained.

Trust-based therapeutic relationships therefore require particular interpretative caution. Humans may perceive a relationship as founded on reciprocity and security, whereas the non-human animal may experience it primarily as a controlled social environment. Consequently, the analysis of trust in interspecies relationships requires simultaneous consideration of social, psychological, ethological and biological perspectives.

## 7. Applied and Boundary Contexts of Interpretative Asymmetry

The analysis of therapeutic human–animal relationships becomes particularly meaningful when asymmetry is examined in concrete contexts. In this review, applied AAI/AAT contexts are distinguished from cultural boundary narratives. Dogs and horses are discussed as common domesticated species participating in animal-assisted interventions, whereas selected wild-animal narratives are treated only as boundary examples of projection and romanticization. This distinction responds to the need to separate empirical welfare concerns from cultural interpretations of interspecies relationships.

### 7.1. Dogs in Animal-Assisted Interventions: Benefit, Interpretation, and Welfare

Dogs are among the most frequently involved non-human animals in animal-assisted interventions because of their social responsiveness to humans, trainability and long domestication history. In therapeutic contexts, behaviours such as approaching a patient, remaining still during touch, following the handler, gazing at a human or tolerating close contact may be interpreted as signs of affection, trust or willingness to participate. These interpretations may be valid in some contexts, but they should not be assumed without behavioural and welfare assessment.

The dog’s role in AAI/AAT makes interpretative asymmetry particularly visible. The patient or therapist may experience the dog as emotionally available, calming and supportive, while the dog’s experience may involve a mixture of affiliation, habituation, handler orientation, fatigue or attempts to reduce social tension. Because therapy dogs are often expected to remain calm and predictable in unfamiliar environments, behavioural inhibition may be misread as comfort. Subtle indicators such as lip licking, yawning, gaze aversion, body tension, reduced exploration, seeking proximity to the handler or avoidance of contact should therefore be interpreted in context rather than dismissed as irrelevant.

For this reason, canine-assisted interventions require welfare protocols that include session limits, rest breaks, access to water, protection from excessive handling, the possibility of withdrawal and careful handler attention to behavioural signals. The handler should not act solely as a facilitator of human therapeutic benefit but also as an advocate for the dog. This is especially important in work with children, hospital patients or groups, where unpredictability, noise, repeated touching and prolonged exposure may increase the dog’s workload. The core ethical problem is therefore not whether dogs can benefit humans, but whether human benefit is evaluated together with the dog’s agency and welfare.

### 7.2. Horses in Equine-Assisted Interventions: Welfare, Load, and Control

Horses represent another major group of non-human animals involved in therapeutic and supportive practices, including therapeutic riding, hippotherapy, equine-assisted psychotherapy and equine-assisted learning. A full review of equine-assisted interventions lies beyond the scope of this article; however, their inclusion is necessary because they reveal a distinctive form of asymmetry in which human therapeutic benefit may be closely linked to the animal’s body, movement and behavioural regulation.

In equine-assisted contexts, the horse may be interpreted by humans as calm, accepting, responsive or emotionally attuned. At the same time, the horse may be exposed to specific welfare challenges, including biomechanical load, rider imbalance, abnormal pressure, repeated mounting and dismounting, handler control, environmental unpredictability and repeated sessions with different participants. In therapeutic riding or hippotherapy, the physical condition of the rider and the therapeutic goals of the session may influence the horse’s musculoskeletal and emotional workload. In ground-based interventions, welfare concerns may involve forced proximity, repeated grooming, leading exercises, exposure to emotionally intense human behaviour or limited opportunity to avoid interaction.

These examples do not imply that equine-assisted interventions are inherently harmful. Rather, they show that horses should not be treated as neutral therapeutic surfaces or symbolic mirrors for human emotion. Welfare assessment should include behavioural indicators, posture, locomotor comfort, signs of pain or stress, recovery time, workload distribution and the horse’s possibility of withdrawal. From the perspective of interpretative asymmetry, the central question is whether the horse’s participation is evaluated through human therapeutic outcomes alone or through a combined assessment of human benefit and equine welfare.

### 7.3. Cultural Boundary Examples: Projection Beyond Therapeutic Settings

The following examples are not presented as cases of animal-assisted therapy in the strict sense, nor are they treated as empirical evidence of therapeutic efficacy or animal welfare outcomes. Encounters with wild species, such as an octopus, bears or wolves, do not meet the definitional criteria of AAI/AAT adopted in this review. They are included only as cultural boundary narratives that make visible the broader interpretative mechanisms analyzed here: self-healing narratives, projection, romanticization and the tendency to assign human relational categories to non-human animal behaviour without direct access to the animal’s perspective. Their analytical value lies precisely in their distance from structured therapeutic practice: they show how far human meaning-making may extend when the animal’s experience remains uncertain or unverifiable.

My Octopus Teacher can be understood as a boundary narrative of human healing through contact with a wild non-human animal. The documentary portrays the octopus as profoundly meaningful for the human protagonist’s emotional recovery, but the animal’s perspective remains accessible only through human observation and interpretation. Research on cephalopod cognition supports the view that octopuses possess complex behavioural flexibility and learning capacities [38,39,40,41], but such capacities do not justify unambiguous attribution of human-like emotional reciprocity. The example illustrates how a relationship perceived as therapeutic for the human may remain uncertain from the perspective of the wild animal’s welfare.

Grizzly Man and The Wolfman [42] represent more extreme boundary examples rather than therapeutic relationships. In both cases, humans interpret close contact with wild carnivores through narratives of trust, belonging or exceptional interspecies understanding. Ethological evidence concerning bears and wolves, however, indicates that tolerance of human presence may reflect habituation, risk assessment or context-dependent social behaviour rather than reciprocal human-like bonding [43,44,45,46]. These examples show the limits of romanticizing interspecies relationships and the risks that arise when human meanings override species-specific behavioural realities.

Table 3 compares three contexts through which interpretative asymmetry becomes visible. It no longer treats cultural examples as the main case studies of therapy, but places them alongside applied canine and equine contexts to clarify the difference between structured AAI/AAT and boundary narratives of projection and romanticization.

By placing these contexts side by side, Table 3 does not equate structured AAI/AAT with wild-animal narratives. Instead, it shows that different contexts reveal different forms of the same underlying problem: human interpretation may obscure the non-human animal’s welfare status, agency, bodily load or epistemic opacity. This distinction allows the article to retain the cultural examples as boundary cases while keeping therapeutic practice and animal welfare as the applied focus of the analysis.

## 8. Discussion

Therapeutic human–animal relationships have long been interpreted primarily through the benefits they provide to humans. Non-human animals are commonly perceived as sources of emotional support, psychological stability, social motivation or improved quality of life. However, the analysis presented in this article demonstrates that these relationships are considerably more complex and cannot be understood solely in terms of their therapeutic effectiveness for humans.

The central conceptual contribution of this review is the clarification of interpretative asymmetry as a structural feature of therapeutic interspecies relationships. This asymmetry does not mean that humans and non-human animals cannot form meaningful relationships. Rather, it means that humans usually possess the dominant interpretative position: they define the purpose of the interaction, organize the environment, determine acceptable behaviour and interpret the non-human animal’s responses. The non-human animal participates actively, but its perspective remains mediated through human observation and interpretation.

This issue does not imply that non-human animals are incapable of forming social bonds or responding emotionally to the presence of humans. Contemporary ethology and neurobiology provide substantial evidence that animals can participate in complex social interactions, respond to emotional signals and establish long-lasting affiliative relationships [4,6]. At the same time, the biological mechanisms regulating animal social behaviour do not necessarily correspond to human understandings of emotional attachment.

AAI/AAT should therefore be understood not as a separate topic replacing the central focus of the article, but as a particularly important context in which interpretative, communicative, structural and welfare-related asymmetries become visible. The fact that a non-human animal supports human emotional regulation or motivation does not automatically demonstrate that the animal experiences the interaction as positive, reciprocal or voluntary. Human benefit must be interpreted together with behavioural, physiological and contextual indicators of animal welfare.

The concept of affective labour helps to articulate this problem. Non-human animals in therapeutic and supportive settings may provide comfort, emotional regulation, social facilitation and a sense of safety. These contributions may be highly valuable for humans, but they also constitute expectations placed on non-human animals. When the animal’s affective labour becomes naturalized as “what the animal does”, its workload, fatigue, agency and limits may become invisible. This is especially important when the animal is described as a partner while the conditions of participation remain almost entirely human-controlled.

The present review also has practical implications for the organization of animal-assisted interventions. No single universal protocol can be applied identically to all species, individuals and therapeutic contexts; however, the absence of a universal protocol does not remove the need for minimum welfare safeguards. Assessment should begin with the individual non-human animal, including its temperament, age, physiological condition, previous experience, emotional state and species-specific needs. Workload limits, rest breaks, predictable routines and the possibility of withdrawal from interaction should be treated as core elements of ethical AAI/AAT practice rather than optional additions. These principles translate the conceptual problem of asymmetry into practical responsibility: if the animal’s experience is not directly accessible, the conditions of participation must be designed to make stress, fatigue and refusal more visible.

In this context, the handler should act not only as a facilitator of human therapeutic benefit but also as an advocate for the non-human animal. This role requires more than technical control of the session. It involves continuous observation of behavioural signals, protection from excessive contact, recognition of motivational conflict and readiness to interrupt or modify the interaction when welfare indicators suggest discomfort. Regular supervision or consultation with an independent expert in animal behaviour and welfare may help prevent the gradual normalization of stress signals and support more accurate interpretation of the animal’s needs. Such external support is particularly important when the same animal repeatedly works with vulnerable human participants, children, emotionally intense situations or institutional environments, contexts in which ethical responsibilities toward both children and therapy animals have been emphasized [47]. Early ethical discussions of AAI/AAT already emphasized that the welfare of the animal cannot be treated as secondary to human benefit [48].

Accurate recognition of behavioural and physiological stress signals protects not only the non-human animal but also the human participant. Misinterpreting avoidance, inhibition, fatigue or stress as calmness, trust or attachment may increase the risk of incidents, reduce therapeutic quality and weaken the ethical foundation of the intervention. Conversely, a welfare-sensitive approach can improve both safety and relational quality, because the animal is not forced to function as a silent therapeutic instrument. In this sense, animal welfare is not external to therapeutic efficacy but forms part of a One Welfare perspective linking animal well-being, human safety and the ethical quality of care [49].

The cultural boundary examples discussed in this review further demonstrate the human tendency to romanticize relationships with non-human animals, particularly in the context of wild nature. Such narratives are useful not because they provide evidence about AAI/AAT, but because they reveal the broader interpretative mechanisms through which humans project meanings onto animal behaviour. They therefore support the conceptual argument while remaining clearly separated from empirical welfare evidence.

Future research should focus on developing methods that allow for a more objective and multidimensional assessment of the experiences of non-human animals participating in therapeutic relationships. Particularly important are mixed-methods studies integrating behavioural ethograms, physiological indicators such as cortisol and heart rate variability, handler reports, environmental context and long-term welfare outcomes. A better understanding of these processes may contribute to the development of more ethical and genuinely welfare-sensitive models of human–animal therapeutic relationships.

## 9. Limitations of the Study

This article is a narrative and interdisciplinary review rather than a systematic review or meta-analysis. It did not follow a PRISMA-based protocol, did not apply a formal risk-of-bias assessment and does not provide a quantitative synthesis, effect-size estimates or statistical verification of the claims advanced. Its conclusions are therefore conceptual and thematic rather than statistically generalizable.

The concept of interpretative asymmetry is used here as a theoretical and analytical category, not as a directly measured empirical variable. The article does not claim to quantify asymmetry or to determine the subjective emotional states of individual non-human animals. Instead, it identifies mechanisms through which human interpretation, communicative limitations, institutional control and asymmetries of agency may shape therapeutic relationships.

The cultural examples discussed in the manuscript are illustrative boundary narratives rather than empirical evidence of animal-assisted therapy or animal welfare outcomes. They are included to show how projection, romanticization and human-centred meaning-making may operate in interspecies narratives. Consequently, they should not be understood as evaluations of the specific animals involved or as evidence for the welfare effects of the relationships portrayed.

A further limitation is the heterogeneity of the AAI/AAT literature. Studies differ in species, settings, populations, terminology, intervention protocols and welfare measures. Future empirical research should therefore combine qualitative analysis of human interpretation with standardized behavioural and physiological indicators in non-human animals participating in real therapeutic settings. Such research is needed to move from conceptual recognition of asymmetry toward operational tools for identifying when therapeutic interaction supports welfare and when it produces overload, inhibition or constrained participation.

## 10. Conclusions and Future Research Directions

Therapeutic human–animal relationships constitute complex social interactions whose significance extends beyond traditional understandings of animal-assisted interventions. The analysis presented in this article demonstrates that the interpretation of such relationships is largely based on human understandings of communication, emotions and reciprocity.

Ethology, neurobiology and animal welfare science indicate that non-human animals participating in therapeutic relationships are not passive elements of the therapeutic environment but active participants in social interactions. At the same time, humans continue to possess only limited capacity to fully recognize the emotional experiences of non-human animals and the significance that such relationships may hold for other species.

The analysis of therapeutic human–animal relationships requires the integration of humanistic reflection with biological and ethological knowledge. Only an interdisciplinary approach makes it possible to acknowledge the significance of these relationships for humans while simultaneously considering the welfare, needs and biological limitations of the non-human animals involved.

Future research should move toward mixed-methods designs that combine qualitative assessment of human interpretations with quantitative welfare indicators in non-human animals, including behavioural ethograms, cortisol, heart rate variability, workload measures and long-term follow-up. Such research may help determine when therapeutic interaction supports welfare and when it produces overload, inhibition or constrained participation.

Ultimately, recognizing the non-human animal not as a passive therapeutic instrument but as an active, interpreting and welfare-sensitive participant changes the ethical and scientific understanding of therapeutic interspecies relationships. The effectiveness of such relationships should not be evaluated solely through human benefit, but also through the animal’s agency, welfare, workload and capacity to withdraw from or regulate interaction.

## Figures and Tables

**Table 1:** Examples of interpretative asymmetry between common human readings and alternative ethological readings of non-human animal behaviour.

Observed Behaviour/Situation	Common Human Reading	Alternative or Overlooked Ethological Reading
Approach or proximity-seeking	Affection, trust or attachment	Contact-seeking, learned expectation, social referencing or reward anticipation
Remaining still or quiet	Calm acceptance	Inhibition, uncertainty, fatigue or passive coping
Yawning, lip licking or head turning	Patience, gentleness or relaxation	Appeasement, tension regulation or mild stress signalling
Sustained gaze or following the handler	Bonding, loyalty or focused attention	Monitoring, handler orientation, arousal or learned response
Tolerance of touch or close contact	Comfort, consent or emotional closeness	Habituation, learned tolerance, constrained choice or limited withdrawal
Withdrawal, avoidance or low responsiveness	Disinterest, stubbornness or serenity	Discomfort, overload, fatigue or need for distance

**Table 2:** Welfare-supporting conditions and welfare risks in therapeutic relationships involving non-human animals.

Domain	Welfare-Supporting Condition	Welfare Risk If Poorly Managed
Social contact	Voluntary affiliative interaction	Excessive exposure, forced proximity or social fatigue
Environment and routine	Predictability, safety and species-appropriate setting	Sensory overload, monotony or environmental stress
Workload and recovery	Session limits, rest periods and recovery time	Fatigue, cumulative load or chronic stress
Autonomy and control	Choice, consent cues and possibility of withdrawal	Constrained choice, learned inhibition or passive coping
Handler role	Skilled observation, advocacy and protection	Misreading of signals or inappropriate therapeutic demands
Welfare monitoring	Behavioural indicators and, where feasible, physiological measures	Invisible overload or delayed recognition of discomfort

**Table 3:** Applied and boundary contexts through which interpretative asymmetry becomes visible.

Context	Human-Centred Reading	Non-Human Animal/Welfare Issue	Main Asymmetry
Dog-assisted interventions	Comfort, trust, attachment or cooperation	Subtle stress signals, fatigue, handler orientation or limited withdrawal	Interpretative and welfare-visibility asymmetry
Equine-assisted interventions	Calmness, mirroring, therapeutic movement or emotional attunement	Biomechanical load, pain, cumulative workload, recovery and control of movement	Welfare, bodily-load and agency asymmetry
Cultural boundary narratives	Exceptional bond, healing, belonging or transcendence of species boundaries	Animal perspective remains inferential or unverifiable; projection, habituation or disturbance may be overlooked	Projection and epistemic asymmetry

## Data Availability

No new data were created or analyzed in this study. Data sharing is not applicable to this article.
